# Pattern of recovery following total shoulder arthroplasty and humeral head replacement

**DOI:** 10.1186/1471-2474-15-306

**Published:** 2014-09-18

**Authors:** Helen Razmjou, Paul Stratford, Deborah Kennedy, Richard Holtby

**Affiliations:** Holland Orthopaedic & Arthritic Centre, Sunnybrook Health Sciences Centre, 43 Wellesley Street East, Toronto, Ontario M1Y 1H1 Canada; Department of Physical Therapy, Faculty of Medicine, University of Toronto, Toronto, Canada; Sunnybrook Research Institute, Sunnybrook Health Sciences Centre, Toronto, Canada; School of Rehabilitation Science, McMaster University, Hamilton, Canada; Department of Clinical Epidemiology & Biostatistics, McMaster University, Hamilton, Canada; Division of Orthopaedic Surgery, Department of Surgery, Faculty of Medicine, University of Toronto, Toronto, Canada

**Keywords:** Total Shoulder Arthroplasty, Humeral Head Replacement, Disability recovery pattern

## Abstract

**Background:**

Understanding the pattern of recovery and expected rate of change after shoulder arthroplasty is helpful to clinicians and patients for setting realistic expectations and goals. The purpose of this study was to describe the pattern of recovery over a 2-year period for patients receiving either a Total Shoulder Arthroplasty (TSA) or Humeral Head Replacement (HHR).

**Methods:**

This was a secondary analysis of prospectively collected data of patients who had undergone TSA or HHR and were followed for up to 2 years. Patients were seen prior to surgery and at 6 months, one year and two years after surgery and completed the American Shoulder and Elbow Surgeon’s (ASES) questionnaire, Relative Constant Murley score (RCMS) and underwent range of motion and strength assessment.

**Results:**

Data of 134 patients who had surgery from April 2001 to July 2011 were used for analysis. One hundred and eight patients underwent TSA (81%) and 26 (19%) had HHR. Both surgeries were associated with a statistically significant improvement in physical symptoms, ASES, RCMS, range of motion and strength (p <0.0001). The greatest change for all outcomes occurred within the first 6-months of surgery. Improvement in ASES, RCMS continued up to 12-months and then plateaued. Improvement in physical symptoms leveled off at 6-months in the HHR group but continued up to 12-months in the TSR group. Strength showed improvement in both groups up to 24-months post-surgery.

**Conclusion:**

Both TSA and HHR groups showed a statistically significant improvement in perceived disability, range of motion and strength over two years with the greatest improvement made by 6 months. The recovery profiles for the surgeries showed different patterns.

**Electronic supplementary material:**

The online version of this article (doi:10.1186/1471-2474-15-306) contains supplementary material, which is available to authorized users.

## Background

Primary osteoarthritis of the glenohumeral joint is characterized by loss of the articular cartilage producing joint space narrowing, subchondral sclerosis and osteophyte formation. Surgical management is considered when conservative treatment such as rehabilitation, intra-articular corticosteroid injection, anti-inflammatory medication and analgesics fail to provide relief.

Traditionally, Total Shoulder Arthroplasty (TSA) is the treatment of choice for primary and posttraumatic osteoarthritis and also some inflammatory conditions of the shoulder as long as the rotator cuff remains intact. Humeral Head Replacement (HHR) has also been utilized for primary osteoarthritis, but is more often indicated for patients with inflammatory arthritis with severe rotator cuff deficiency, cuff tear arthropathy, proximal humeral fractures, osteonecrosis of the humeral head with a preserved glenoid, or in the presence of insufficient glenoid bone stock[1]. The literature shows that the majority of patients improve significantly with respect to pain and range of motion following TSA[2, 3]. The outcome of HHR seems to be less predictable[4–6] partly due to rotator cuff or glenoid bone deficiency. To eliminate the impact of cuff pathology or glenoid co-morbidity, a number of randomized clinical trials with homogenized samples (patients with primary osteoarthritis) have been conducted to better examine the difference in outcome of these two procedures[6, 7]. These studies showed that total shoulder arthroplasty provided better functional outcomes than hemiarthroplasty for patients with primary osteoarthritis of the shoulder.

Overall, the inferior outcome of HHR is known and partly justified due to more extensive pathology of the shoulder in this population. However, the limitation of the available research is lack of information on important time points which helps to predict when patients reach their maximal recovery as measured by patient reported measures of pain and disability or functional abilities including range of motion and strength.

We are not aware of any studies that have determined the specific time point of maximal recovery following shoulder arthroplasty procedures. Understanding the pattern of change would help clinicians to communicate the expected results of surgery and time frame for reaching maximal recovery to their patients. It is also important that studies are controlled for sex and age as aging affects the overall function and females have shown less improvement after shoulder surgery related to rotator cuff pathology[8] and lower extremity arthroplasty[9–11].

The purpose of this study was to describe the pattern of recovery over a 2-year period for patients receiving either a Total Shoulder Arthroplasty (TSA) or Humeral Head Replacement (HHR).

## Methods

This was a secondary analysis of prospectively collected data of patients with advanced osteoarthritis of the glenohumeral joint who had undergone TSA or HHR and had participated in previous formal studies and were followed up for up to 2 years. This study has adhered to the STROBE guidelines for observational studies and has received ethics approval from the Research Ethics Board of the Sunnybrook Health Sciences Centre.

### Subjects

The inclusion criteria included age greater than 18 years, a diagnosis of advanced primary osteoarthritis or inflammatory arthritis with or without rotator cuff pathology that had not responded to conservative treatment. The exclusion criteria included inability to speak or read English, evidence of infection, underlying metabolic disease, avascular necrosis, or capsulorraphy arthropathy. Patients with primary osteoarthritis of the glenohumeral joint and an intact rotator cuff underwent TSA. Those with humeral fractures and a normal glenoid articular surface or with severe glenoid deficiency with or without cuff tear arthropathy received HHR. In addition, younger patients and those with significant medical co-morbidities underwent HHR, rather than reverse arthroplasty, as the reverse prosthesis may not be sufficiently durable in younger age group and may have higher complication rate in the presence of other medical conditions.

All patients followed a standardized rehabilitation protocol. A sling was used for two weeks with immediate active assisted mobilization following surgery. Sub-maximal isometric exercises started at four weeks post-operatively. Active exercises started at 6 weeks in lying progressing to upright position at 7 weeks. Resistive exercises involving theraband started at 8-10 weeks. Exercises related to internal rotation followed the other directions of movement with a 4-week delay to avoid strain on subscapularis.

### Outcome measures

Two patient-oriented outcome measures and a record of physical symptoms were obtained 2-3 weeks before surgery and on clinic visits at 6, 12, and 24 months after surgery. The outcome measures were the American Shoulder and Elbow Surgeon’s (ASES) assessment form[12] and the Relative Constant Murley score (RCMS)[13]. The ASES is a 100-point scale, 50 points of which are derived from patient self-report of pain on a visual analog scale and 50 points of which are computed from a formula using the cumulative score of 10 activities of daily living derived using a four-point ordinal scale. The Constant Murley score is a combined measure, containing a patient reported component (35%) and the clinical assessment of range of motion and strength (65%). The absolute score is then converted to the relative score by accounting for age and sex-related differences. Higher scores on the ASES and RCMS indicate less disability. Both measures have established validity and reliability in patients with glenohumeral osteoarthritis[14, 15]. Physical symptoms were measured by the physical symptoms domain of the Western Ontario Osteoarthritis Shoulder (WOOS) Index[16]. Symptoms captured pain with movement, nagging constant pain, weakness, stiffness, grinding and impact of weather changes on pain. The score of the physical symptoms domain varied from 0 as no pain to 600 as the maximal pain and discomfort. Performance measures included active range of motion (ROM) in 3 directions (flexion, abduction, external rotation in neutral, and hand behind back) and strength in scapular plane elevation. Strength was measured with a simple tensiometer with the shoulder at 90 degrees of elevation in the plane of the scapula and the elbow extended while the clinician pulled down on the tensiometer. The maximum painfree force that the patient could resist for 5 seconds as the examiner pulled down on the device was measured. In the case of pain while holding the position, strength was given a score of zero.

### Statistical analyses

Descriptive analyses of patients’ characteristics and outcome measures’ summary scores were performed. This study applied a fixed occasion repeated measures design. We applied Generalized Estimating Equations (GEE) to test for differences in the surgical groups’ change trajectories for each outcome measure. GEE fit population averaged panel data models. Complete data for a given outcome measure at all time-points is not required for a patient to be included in the analysis. Dependent variables were the outcome measures assessed at multiple time-points. The independent variables were surgical group (levels were TSA and HHR) and measurement occasion (levels were pre-surgery, 6, 12, 24-months); covariates were gender and age. All analyses were conducted using STATA version 13.0 (STATACorp, College Station, TX).

## Results

Data of 149 patients were reviewed. Three patients died of causes not related to shoulder arthroplasty. Twelve patients (6 in the TSA and 6 in the HHR group) had missed two consecutive assessments, one being the 2 year follow-up, and were not included in the analysis. The baseline characteristics (age, physical symptoms, ASES, RCMS) of these 12 patients were not different than the remaining. Data of 134 patients (73 females (54%), 61 males (46%), mean age = 67 years, SD: 9) who had surgery from April 2001 to July 2011 were used for final analysis. One hundred and eight patients underwent TSA (81%) and 26 (19%) had HHR (Table 1). The diagnoses were primary osteoarthritis (96%) and inflammatory arthritis (4%) with intact rotator cuff in the TSA group. The Neer II prosthesis was used in 18 patients with 49 patients receiving the Bigliani-Flatow (BF) prosthesis and 41 receiving the Total Evolutive Shoulder System (TESS). Seventy percent (18) of the HHR group had primary osteoarthritis with deficient glenoid bone. Four patients had inflammatory arthritis (15%). There were three humeral head fractures (11%) and one cuff tear arthropathy (4%). The patient with cuff tear arthropathy was an 80 year old female who had undergone a rotator cuff repair five year previously and was not a good candidate for reverse arthroplasty due to other medical comorbidities. The majority of the patients in the HHR group received the BF prosthesis (22, 73%) with three TESS prostheses and one Dupuy Global CTA Advantage system. Patients in the TSA group were not different than patients in the HHR group with respect to age, sex, or mechanism of injury (p > 0.05).

**Table 1:** **Baseline data for the Outcome Measures**

HHR				TSR		
Variable	N	Mean	SD	N	Mean	SD
ASES	26	27.08	17.62	108	34.37	15.97
ACMS	26	20.60	11.4	106	21.10	11.50
Pain*	26	1.70	2.80	108	1.50	2.50
ADL*	26	6.1	3.2	108	6.9	2.7
ROM*	26	9.20	6.50	106	8.8	6.1
Strength*^§^	26	3.60	4.22	108	4.02	3.83
RCMS (%)	26	27.48	14.85	106	27.83	14.32
PS	26	457.81	101.04	108	410.04	107.75
Flex	26	65.88	29.70	108	63.89	27.26
Abd	26	50.88	27.13	108	44.02	21.69
Ext Rot	26	14.23	13.39	108	12.64	10.97

*:Components of the Constant Murley score.

^§^: In Pounds.

**Figure 1:**
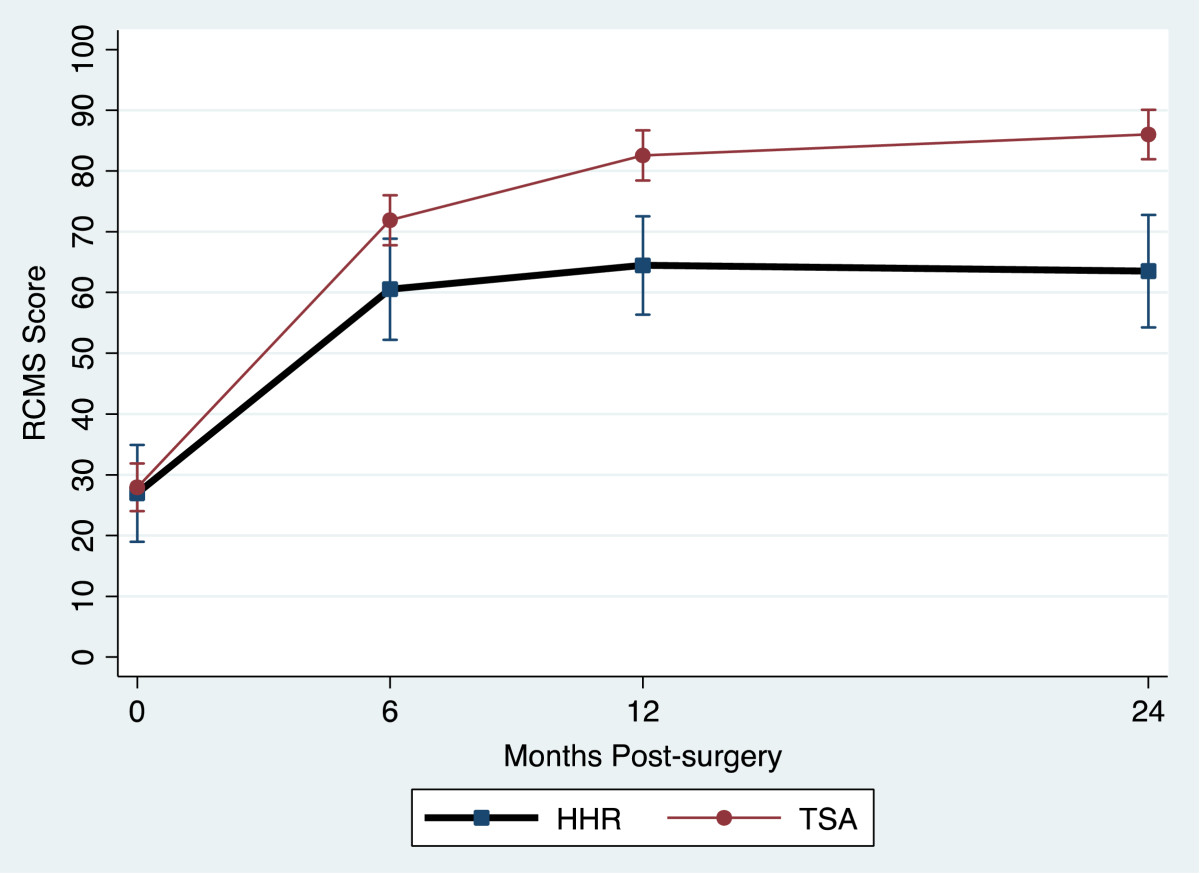
**Relative Constant Murley Score change profile.**

**Figure 2:**
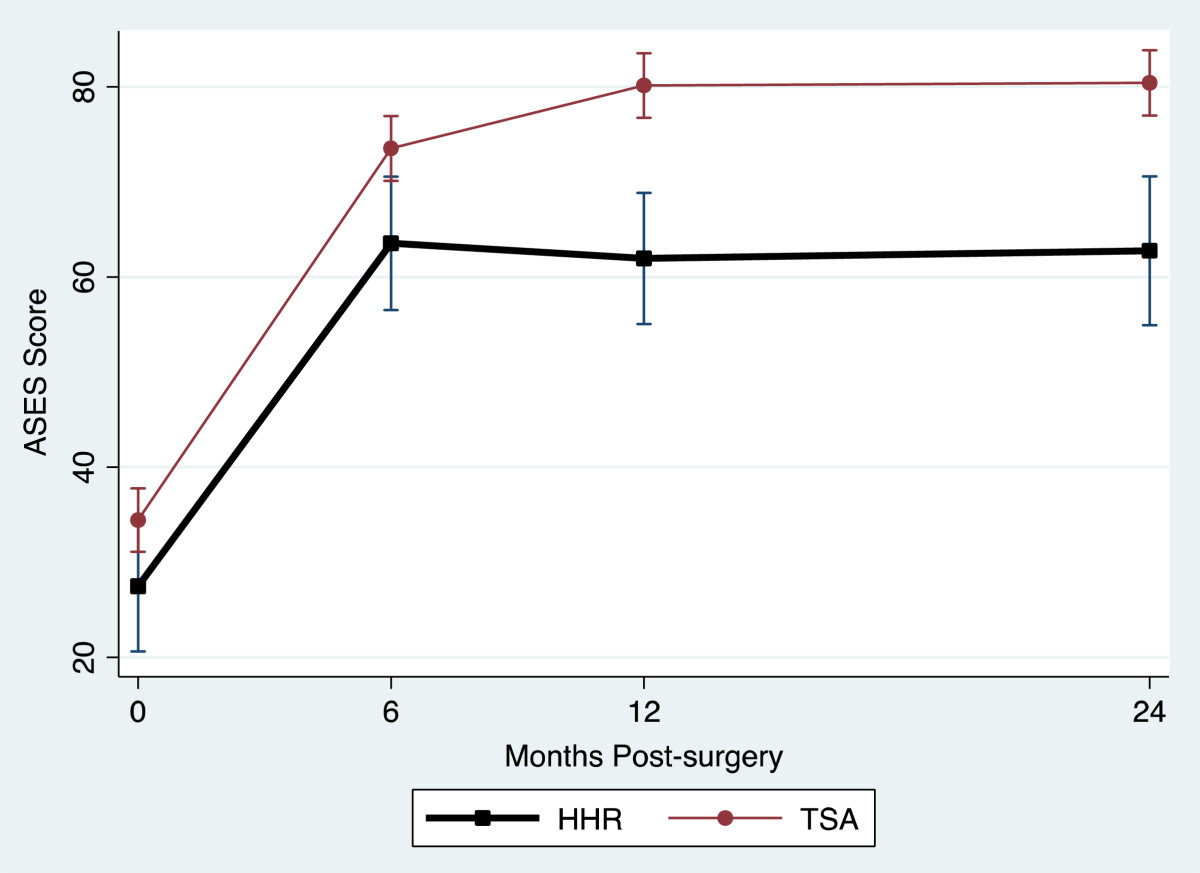
**ASES Score change profile.**

**Figure 3:**
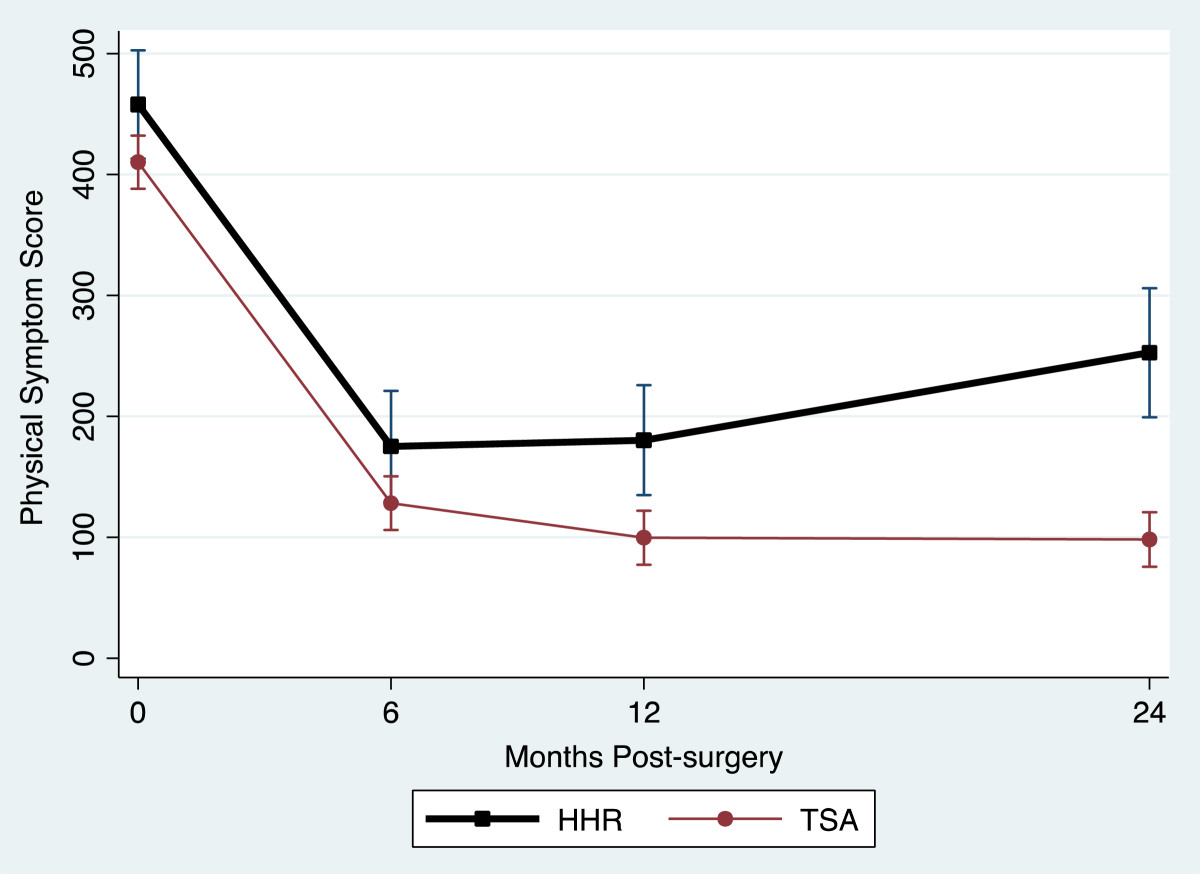
**Physical symptom change profile.**

**Figure 4:**
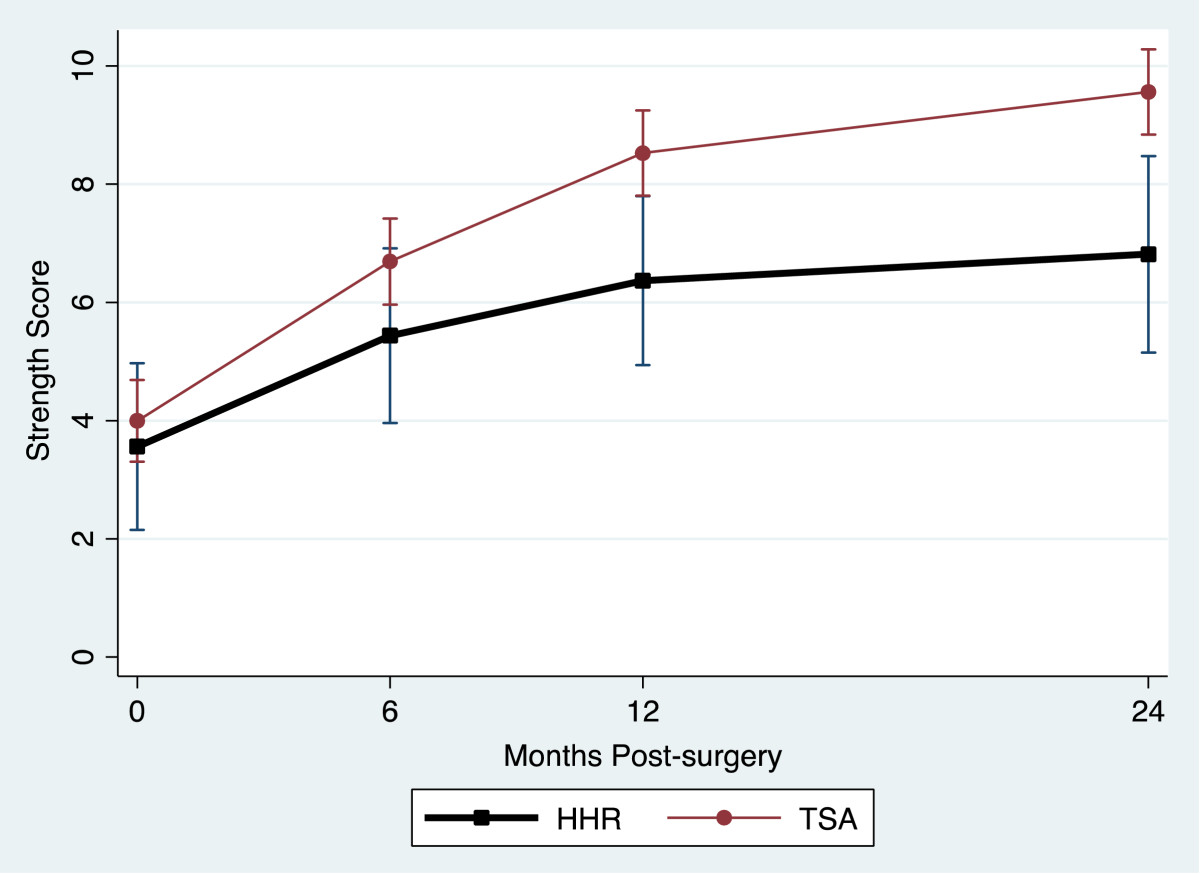
**Strength change profile.**

Table 2 provides a summary of the outcome measure mean values for the TSA and HHR groups for each assessment occasion. All outcome measures reflected large and statistically significant improvements at 2 years. Figure 1 and Figure 2 display the TSA and HHR change profiles for the RCMS and ASES respectively. The greatest change for all outcomes occurred within the first 6-months of surgery. Improvement continued up to 12-months and then plateaued in all measures except physical symptoms, external rotation and strength which showed a different pattern. Figure 3 displays the TSA and HHR change profiles for the physical symptoms. For patients receiving TSA, improvement occurred up to 12-months and then plateaued. For patients receiving HHR, improvement leveled off at 6-months and slightly declined thereafter. Figure 4 shows the change profile for strength which shows improvement in both groups up to 24-months post-surgery.

**Table 2:** **Means of the outcome measures at specific assessment occasions**

Outcome measure	Pre-surgery (95% CI)	6-months (95% CI)	12-months (95% CI)	24-months (95% CI)
ASES				
HHR	27 (21, 34)	63 (56, 70)	62 (55, 69)	63 (55, 70)
TSA	34 (31, 38)	73 (70, 77)	80 (77, 83)	80 (77, 84)
RCMS				
H*HR*	27 (19, 35)	60 (52, 69)	64 (56, 72)	63 (54, 73)
TSA	28 (24, 32)	72 (68, 76)	82 (78, 87)	86 (82, 90)
Physical Symptoms				
HHR	458 (413, 503)	175 (129, 221)	180 (135, 226)	253 (199, 306)
TSA	410 (388, 432)	128 (106, 150)	100 (77, 122)	98 (76, 121)
Strength				
HHR	3.6 (2.1, 5.0)	5.4 (3.9, 6.9)	6.4 (4.9, 7.8)	6.8 (5.1, 8.5)
TSA	4.0 (3.3, 4.7)	6.7 (6.0, 7.4)	8.5 (7.8, 9.2)	9.6 (8.8, 10.3)
Flexion				
HHR	66 (54, 79)	98 (85, 111)	104 (91, 117)	107 (93, 121)
TSA	64 (57, 70)	116 (110, 123)	130 (123, 136)	131 (125, 137)
Abduction				
HHR	51 (38, 64)	83 (70, 97)	93 (80, 107)	84 (71, 98)
TSA	44 (38, 51)	101 (94, 107)	115 (109, 122)	119 (112, 125)
Ext. Rot.				
HHR	14 (7.9, 20)	40 (33, 46)	40 (34, 46)	42 (35, 49)
TSA	13 (10, 16)	42 (39, 45)	47 (44, 50)	47 (44, 50)

Change over time (2-year scores - pre-op scores) was significant for TSA and HHR groups for all measures at p < 0.0001.

## Discussion

In the present study, we found that both TSA and HHR groups improved in their physical symptoms, disability level and range of motion over time. However, the HHR group reported overall higher disability as measured by the ASES, RCMS, and symptoms. As apparent in the figures, different patterns of recovery were observed in these measures.

Recovery of external rotation was similar between groups. Improvement of external rotation in both groups may be related to change in biomechanics of the replaced joint regardless of type of procedure. It has been shown that an anatomical reconstruction of the humeral head can restore the physiological motions and limited eccentric loading of the glenoid, which may explain more improvement in external rotation than elevation[17].

Although the rate of change was different between HHR and TSA, the most significant recovery of disability, physical symptoms and range of motion was made by 6 months. In terms of the ASES and RCMS outcome measure scores and range of motion, recovery continued to 12 months and then began to plateau. This was also the case for the TSA group with respect to the change profile for the physical symptoms. In contrast, physical symptoms for the HHR group improved till 6 months and then began to decline. A different pattern of recovery was noted for strength which showed continued improvement until 24 months post surgery.

Our results are consistent with the literature which indicates an overall improvement in pain and disability following shoulder arthroplasty[2–7, 18–23]. The contribution of our study is exploring the typical trajectory of recovery after each procedure. This information is useful to clinicians as it enables them to communicate the expected point of maximal recovery or plateau to their patients more accurately. It is also of importance to patients as it helps them to have realistic expectations and set appropriate goals throughout their recovery. More specifically, patients who undergo HHR may benefit from knowing that although elevation may have a slower and less significant improvement, external rotation, and overall disability will show improvement up to one year with strength gains of up to two years.

### Limitations

Despite consistency between our results and the available literature, the present study involved secondary analysis of prospectively collected data of patients operated by a senior orthopedic surgeon in an academic centre, which may limit generalizability of our findings.

In the present study, most of the recovery happened in the first 6 months. Although shoulder joint replacement is a major surgery with a prolonged recovery, future work might include more sampling prior to the 6-month time period.

## Conclusion

Our study showed a statistically significant improvement in all patient reported and performance measures regardless of the type of arthroplasty. The greatest change for all outcomes measured occurred within the first 6-months of surgery. In the HHR group, despite a slight decline in physical symptoms after 6 months, there was a significant overall improvement from the pre-operative level. Strength continued to improve up to 24 months post surgery. The pattern of recovery differed by outcome measure and type of procedure. Knowledge of these recovery patterns will help clinicians direct appropriate rehabilitation and patients set more realistic goals and expectations.

## Authors’ original submitted files for images

Below are the links to the authors’ original submitted files for images. Authors’ original file for figure 1 Authors’ original file for figure 2 Authors’ original file for figure 3 Authors’ original file for figure 4

## Abbreviations

Abd: Abduction
ACMS: Absolute Constant Murley Score
ADL: Activities of daily living
ASES: American Shoulder and Elbow Surgeon’s form
BF: Bigliani-Flatow
Ext Rot: External Rotation
Flex: Flexion
GEE: Generalized Estimating Equations
HHR: Humeral Head Replacement
PS: Physical Symptoms
ROM: Range of Motion
RCMS: Relative Constant Murley Score
TESS: Total Evolutive Shoulder System
TSA: Total Shoulder Arthroplasty.
