# Supplementary figures and images for: Pattern of recovery following total shoulder arthroplasty and humeral head replacement
Source: BMC Musculoskelet Disord. 2014 Sep 18;15:306. doi: 10.1186/1471-2474-15-306 (PMC4190476; doi:10.1186/1471-2474-15-306)

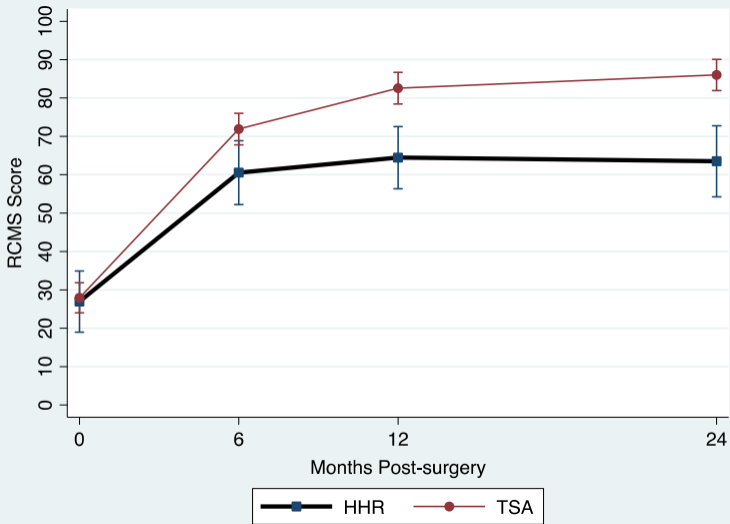

Supplement: Supplementary file 1 — Authors’ original file for figure 1 [file 12891_2014_2253_MOESM1_ESM.pdf]

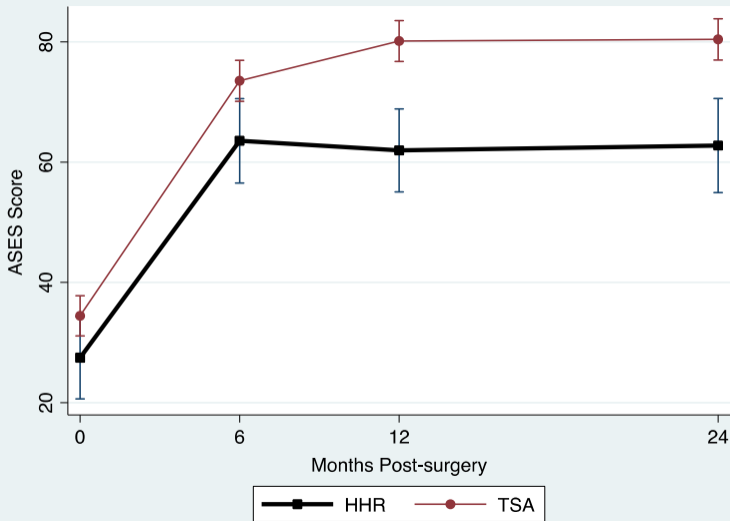

Supplement: Supplementary file 2 — Authors’ original file for figure 2 [file 12891_2014_2253_MOESM2_ESM.pdf]

Physical Symptom Score

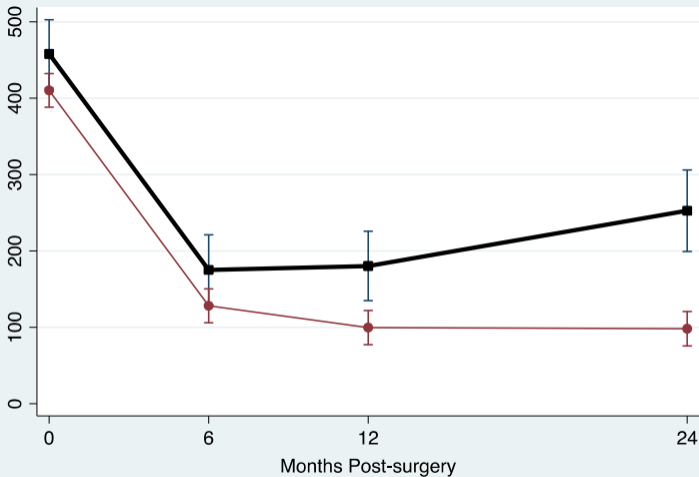

—■— HHR —●— TSA

Supplement: Supplementary file 3 — Authors’ original file for figure 3 [file 12891_2014_2253_MOESM3_ESM.pdf]

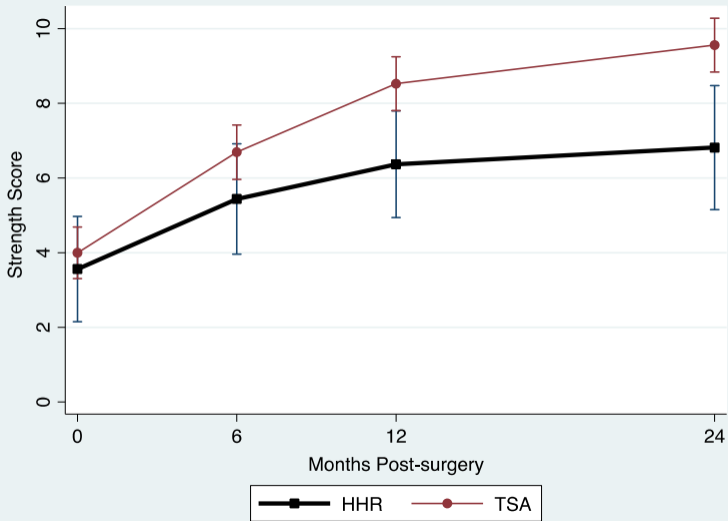

Supplement: Supplementary file 4 — Authors’ original file for figure 4 [file 12891_2014_2253_MOESM4_ESM.pdf]
